# *Rickettsia felis, Bartonella henselae,* and *B. clarridgeiae,* New Zealand

**DOI:** 10.3201/eid1005.030986

**Published:** 2004-05

**Authors:** Patrick J. Kelly, Natalie Meads, Anita Theobald, Pierre-Edouard Fournier, Didier Raoult

**Affiliations:** * Massey University, Palmerston North, New Zealand; † Faculté de Médecine, Marseille, France

**Keywords:** Rickettsia, Bartonella, New Zealand, flea, *Ctenocephalides*

**To the Editor:** The cat flea (*Ctenocephalides felis felis* Bouché, 1935) is a ubiquitous parasite of domestic and wild animals that also feeds readily on people. Recent studies have implicated the cat flea as a vector of new and emerging infectious diseases (*1*). To determine the pathogens in *C. felis* in New Zealand, we collected 3 cat fleas from each of 11 dogs and 21 cats at the Massey University Veterinary Teaching Hospital from May to June 2003. The fleas were stored in 95% alcohol until they were identified by using morphologic criteria and washed in sterile phosphate-buffered saline. The DNA from each flea was extracted individually by using the QiaAmp Tissue Kit (QIAGEN Ltd., Hilden, Germany), according to the manufacturer’s instructions. When polymerase chain reaction (PCR) was performed with primers for *glt*A and *rOmp*A as described (*2*), products were obtained with DNA from 15 (15%) of the fleas. The sequences of the products were identical to those of *Rickettsia felis* (GenBank AF191026) with infected fleas taken from both dogs (3/11; 27%) and cats (7/21; 33%). When PCR was performed with primers for the 16S-23S rDNA interspacer region as described (*3*), products were obtained with DNA of four fleas. The sequences of the products from three fleas (from two cats) were identical to that of *Bartonella henselae* (GenBank AF312495), and the sequence of the product of one flea (from a cat) was identical to that of *B. clarridgeiae* (GenBank AF167989).

Our study is the first to identify *R. felis* in Oceania. The organism is a recently described human pathogen, and infections with this spotted fever group rickettsia have already been reported in 11 persons: 4 persons in the United States, 2 persons in Brazil, 4 persons in Europe, and 1 person in Thailand. The symptoms of the patients were nonspecific and included fever, headache, and rash. Diagnoses were made by sequencing products obtained by PCR with primers for the 17-kDa protein (*4*), citrate synthase (*4*), and PS 120 protein (*5*) genes. *R. felis* has been established in tissue culture (XTC-2 and Vero cells) (*6*), and serologic testing has been used to diagnose infections (*5*). Reports indicate that patients respond rapidly to doxycycline therapy (*5*), and in vitro studies have shown the organism is susceptible to rifampin, thiamphenicol, and fluoroquinolones.

*B. henselae* is an agent of cat-scratch disease, bacillary angiomatosis, bacillary peliosis, endocarditis, bacteremia, and various neurologic and ocular conditions. Cats are the reservoir hosts, and contact with cats and their fleas is an established risk factor for most infections. Although *B. henselae* has been isolated from 17% of domestic cats in New Zealand (*7*), only two human infections have been reported in the country; neuroretinitis was diagnosed in both patients (*8*). In neighboring Australia, however, cat-scratch disease, bacillary angiomatosis, and endocarditis have been diagnosed in numerous patients. Cats are also the reservoir hosts of *B. clarridgeiae* which has been implicated as an agent of cat-scratch disease in humans and aortic valve endocarditis and hepatic disease in dogs (*9*). The organism has been found in cat fleas (as great as 17%) in Europe (*1*), and although we found only one flea infected with *B. clarridgeiae* in New Zealand, this description is the first of the organism in Oceania. However, *B. clarridgeiae* has been found in domestic cats in nearby Indonesia and the Philippines (*10*).

Our findings add to the accumulating data on *R. felis*, *B. henselae,* and *B. clarridgeiae* and should alert medical workers in New Zealand, a common tourist destination, to the possibility that their patients may be infected with these organisms.
